# Inclusion Complexes of a New Family of Non-Ionic Amphiphilic Dendrocalix[4]arene and Poorly Water-Soluble Drugs Naproxen and Ibuprofen

**DOI:** 10.3390/molecules22050783

**Published:** 2017-05-11

**Authors:** Khalid Khan, Syed Lal Badshah, Nasir Ahmad, Haroon Ur Rashid, Yahia Mabkhot

**Affiliations:** 1 Department of Chemistry, Islamia College University, Peshawar 25120, Khyber Pukhtunkhwa, Pakistan; shahbiochemist@gmail.com (S.L.B.); nasirshah121@yahoo.com (N.A.); 2 Department of Chemistry, Sarhad University of Science and Technology, Peshawar 25120, Khyber Pukhtunkhwa, Pakistan; haroongold@gmail.com; 3 Department of Chemistry, College of Science, King Saud University, Riyadh 11495, Saudi Arabia

**Keywords:** amphiphilic calix[4]arene, naproxen, ibuprofen, solubility, inclusion complexes

## Abstract

The inclusion complexes of a new family of nonionic amphiphilic calix[4]arenes with the anti-inflammatory hydrophobic drugs naproxen (NAP) and ibuprofen (IBP) were investigated. The effects of the alkyl chain’s length and the inner core of calix[4]arenes on the interaction of the two drugs with the calix[4]arenes were explored. The inclusion complexes of Amphiphiles **1a**–**c** with NAP and IBP increased the solubility of these drugs in aqueous media. The interaction of **1a**–**c** with the drugs in aqueous media was investigated through fluorescence, molecular modeling, and ^1^H-NMR analysis. TEM studies further supported the formation of inclusion complexes. The length of lipophilic alkyl chains and the intrinsic cyclic nature of cailx[4]arene derivatives **1a**–**c** were found to have a significant impact on the solubility of NAP and IBP in pure water.

## 1. Introduction

**Scheme 1:**
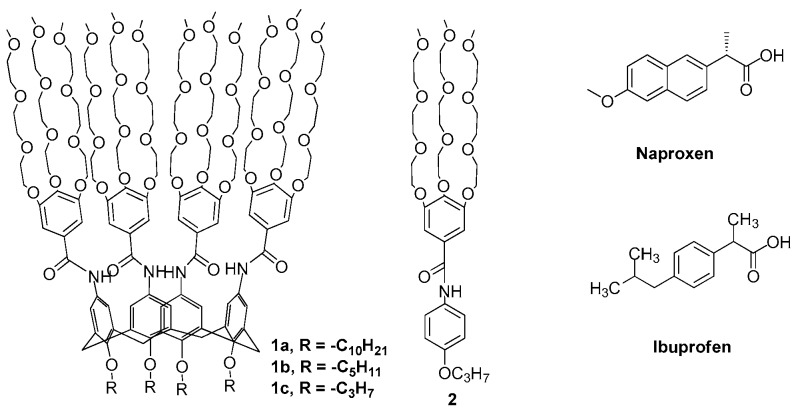
Structures of amphiphilic calix[4]arenes **1a**–**c**, model compound 2, naproxen (NAP), and ibuprofen (IBP).

Naproxen (NAP), (+)-(*S*)-2-(6-methoxynaphthalen-2-yl)propanoic acid, and ibuprofen (IBP), (*RS*)-2-(4-(2-methylpropyl)phenyl)propanoic acid (Scheme 1), are two commercial non-steroid anti-inflammatory drugs (NSAIDs) that are used with increasing frequency as an analgesic and antipyretic; they are prescribed for the treatment of rheumatoid arthritis, osteoarthritis, dysmenorrhea, and other medical conditions [1]. Owing to the poor solubility of NAP and IBP [2], several systems, i.e., chitosan [3], cyclodextrin derivatives [4], and cosolvent systems [5,6,7,8], have been developed to enhance the solubility of drugs in aqueous media in order to help in its better distribution inside the human body [9].

Calix[4]arenes consist of a framework of four phenolic units which are linked together by methylene moieties [10]. These calix[4]arenes are highly attractive to the scientific community due their preparation methods, their suitability as a potential drug carriers, and the ease with which they can cross biomembranes [10]. They have lower side effects and low immunogenicity; as a result, they are encouraging drug carriers in the biomedicine field [11,12,13,14].

Mostly, ionic amphiphilic calixarenes have been employed in the pharmaceutical field as a drug carrier due to their unique biophysical and biochemical properties where they have the ability to take hydrophobic drugs by forming chemical inclusion bodies [15,16,17]. Only in a few cases, however, have non-ionic amphiphilic calixarenes been reported as drug carriers [18]. Here, we report a new family of nonionic amphiphilic calixarenes bearing four tribranched 3,4,5-tris(2-(2-(2-methoxyethoxy)ethoxy)ethoxy)benzamide (3,4,5-TMEEE benzamide) residues at the upper rim and lipophilic alkyl chains at the lower rim [19]. These amphiphilic calix[4]arene derivatives **1a**–**c** significantly increase the solubility of hydrophobic drugs NAP and IBP in pure water through hydrogen bonding and π–π stacking interactions. These interactions reverse the direction of four tribranched 3,4,5-TMEEE benzamide residues, which leads to a change in size and shape of the formed calixarene micelles from solid to hollow forms.

## 2. Results and Discussion

Amphiphilic calix[4]arenes **1a**–**c** and **2** (Scheme 1) were synthesized in high yield (Supplementary Information) and their structures were established on the basis of ^1^H- and ^13^C-NMR, HRMS, and IR spectroscopy. They are soluble in water and are soluble in some organic solvents such as methanol, acetone, THF, chloroform, and benzene but insoluble in non-polar solvents such as *n*-hexane and petroleum ether [19]. The presence of four hydrophilic tribranched 3,4,5-TMEEE benzamide moieties at the upper rim in derivatives **1a**–**c** are expected to trap the water-insoluble drugs through hydrogen bonding and π–π stacking interactions and hence enhance their solubility.

### 2.1. Structure–Solubilization Relationship

The solubility of hydrophobic drugs NAP and IBP in pure water are 0.025 and 0.011 mg/mL, respectively [2], while Amphiphiles **1a**–**c** enhanced the solubility of NAP and IBP considerably in pure water (Table 1). The aqueous solution of calix[4]arene derivatives **1a**–**c** increased the solubility of NAP by a factor of 84, 100, and 116 times, respectively, as compared to its solubility (i.e., 0.025 mg/mL) in pure water. The loading capacity of **1a**–**c** is found to be much higher than the reported value for monoalkyl polyethylene glycol (C_16_H_33_-(OCH_2_CH_2_)_20_OH [2]. Similarly, the solubility of IBP in the aqueous solution of **1a**–**c** was found to be 272, 318, and 345 times larger than its solubility (i.e., 0.011 mg/mL) in pure water [20].

The substituent effect of calix[4]arene on the solubility of NAP and IBP were evaluated by varying the chain length of substituents. It was found that Amphiphile **1c**, with short alkyl chains (propyl), solubilizes larger amount of drugs, as compared to Amphiphile **1a**, with the longest alkyl chains (decyl), demonstrating an inverse relationship between drug solubilizing ability and hydrophobicity of the alkyl group (R) segments of **1a**–**c**.

**Figure 1:**
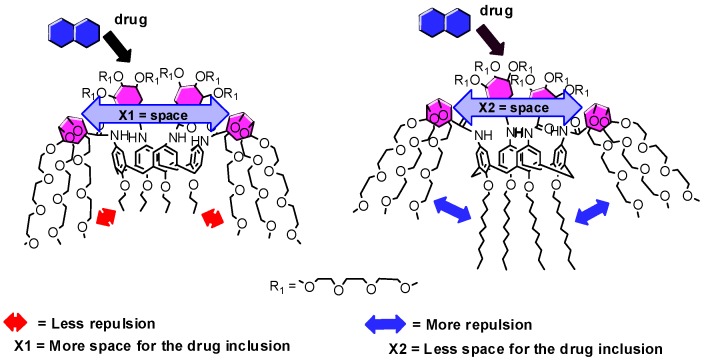
Representation of the relationship between alkyl chain and space for the drug molecule inclusion (X1 > X2).

The impact of alkyl chain length in **1a**–**c** on the solubility of the drugs could be rationalized by considering repulsive forces between the hydrophobic alkyl chains and the four hydrophilic tribranched 3,4,5-TMEEE benzamide substituents of **1a**–**c**. As the length of hydrophobic alkyl chains increases from C_3_ to C_10_, the more repulsive forces develop between the alkyl chains and 3,4,5-TMEEE benzamide substituents, which in turn orient the 3,4,5-TMEEE benzamide substituents in an upward direction and hence minimize space for the drug molecule in the branched 3,4,5-TMEEE benzamide substituents at the upper rim of calixarene (Figure 1).

Furthermore, to establish the role of the intrinsic cyclic core of the calixarene with respect to the solubility of drug in aqueous media, the monomeric analogue **2** was examined. Results showed that aqueous solutions of **2** solubilized only small amounts of the drugs as compared with the solubility of the drugs in the aqueous solutions of Amphiphiles **1a**–**c** (Table 1). The comparison studies indicate that the intrinsic cyclic nature of the calix[4]arene framework is favorable for the NSAID solubilization or the formation of inclusion complexes than that of molecule **2**. Thus, the favorable drug solubilizing ability of **1a**–**c** can be ascribed by taking into account the cyclic nature of the calix[4]arene scaffold, which allows for an organization of the branched 3,4,5-TMEEE benzamide moieties in a well-defined tridimensional architecture with suitable drug solubilizing potential.

### 2.2. Fluorescence Studies

**Figure 2:**
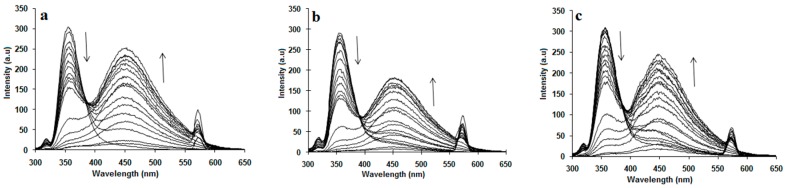
Change in fluorescence spectra of NAP at λ_max_ = 357 nm and of (**a**) **1a**, (**b**) **1b**, (**c**) **1c** at λ_max_ = 450 nm with the concentration of added **1a**–**c**.

Fluorescence measurements manifested the inclusion complexation of **1a**–**c** and NAP in aqueous solutions. The fluorescence spectra associated with the inclusion complexes of **1a**–**c** with NAP were recorded by maintaining a constant concentration for NAP while varying the concentration of **1a**–**c** as shown in Figure 2a–c. Two bands were observed, when **1a**–**c** were excited by a light of 284 nm in water; one strong emission band at 450 nm and second weak emission band at 358 nm. Intramolecular proton- and charge-transfer fluorescence of benzanilide were believed to be responsible for the strong emission band at 450 nm, while the weak emission band at 358 nm was assigned for the normal fluorescence of benzanilide groups [21,22,23].

**Figure 3:**
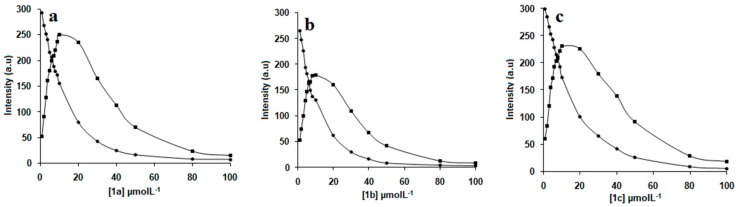
Change in fluorescence intensity of NAP at λ_max_ = 357 (●) and of (**a**) **1a**, (**b**) **1b**, (**c**) **1c** at λ_max_ = 450 nm (￭) with the concentration of added **1a**–**c**.

**Figure 4:**
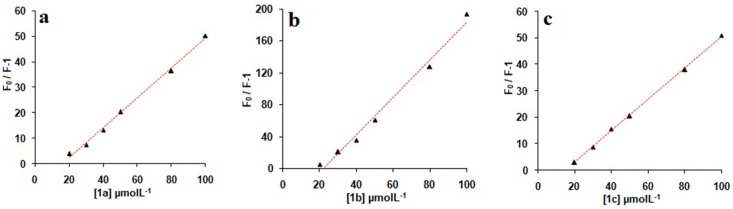
F**_0_**/F-1 value changes with the concentration of added (**a**) **1a**, (**b**) **1b**, (**c**) **1c**. The red straight lines are the fitting results from using the Stern–Volmer equation.

Fluorescence titration of NAP (1.0 × 10^−6^ mol/L) with various concentrations of **1a**–**c** (i.e., 1.0 × 10^−7^ to 1.0 × 10^−4^ mol/L) revealed that the fluorescence intensity of NAP decreased exponentially (Figure 3a–c) with increase in concentrations of **1a**–**c.** The decreases in the fluorescence intensity of NAP were mainly attributed to the inculsion complexation. Linear increase of the fluorescence intensity ratio F_0_/F (the intensity of pure NAP vs. that of mixed NAP with **1a**–**c**) with the concentration of **1a**–**c** in the range of more than 2.0 × 10^−5^ mol/L was well in agreement with the Stern–Volmer equation [24]. Large quenching constant (K_SV_) values of 5.5 × 10^5^, 5.8 × 10^5^, and 6.1 × 10^5^ M^−1^ were obtained for **1a**–**c**, respectively (Figure 4a–c), demonstrating strong interactions between **1a**–**c** and NAP. These results also reveal that quenching constant (K_SV_) values vary inversely with the length of alkyl chains, supporting the assumption that drug solubilizing ability of calix[4]arene derivatives **1a**–**c** is highly dependent on the length of the alkyl chain.

**Figure 5:**
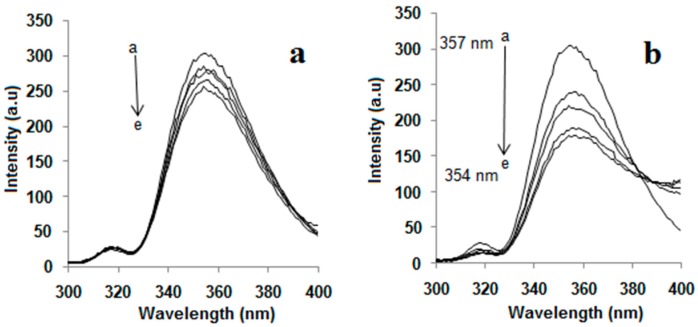
Fluorescence spectra of NAP in the presence of (**a**) **2** and (**b**) **1a**, T = 298 K, [NAP] = 1 × 10^−6^ mol/L = constant. (**a**) **2** (10^−6^ mol/L): (a) 0, (b) 8.0, (c) 7.0, (d) 5.0, (e) 4.0; (**b**) **1a** (10^−6^ mol/L): (a) 0, (b) 8.0, (c) 7.0, (d) 5.0, (e) 4.0.

Furthermore, it was found that the mixing of **1a**–**c** with NAP led to decreases in the fluorescence intensity of NAP with hypsochromic/blueshifts. The hypsochromic/blueshift and the decrease in fluorescence intensity indicated that NAP molecule formed host–guest inclusion complexes with calix[4]arene derivatives **1a**–**c** [25]. Similarly, only a small decrease in fluorescence intensity of NAP upon the addition of monomeric analogue **2** was observed (Figure 5), which indicates a weak interaction between **2** and NAP. It also supports the hypothesis that the intrinsic cyclic nature of calix[4]arene is also fundamental for the solubility of drugs or inclusion complexes.

In order to locate the point of interaction, NAP (2.0 × 10^−4^ mol/L) was mixed with an aqueous solution of 3,4,5-TMEEE benzoic acid (2.0 × 10^−4^ mol/L), the latter also quenched the fluorescence emission of NAP. It confirms that the interaction between NAP and **1a**–**c** should be ascribed neither to the calixarene skeleton itself nor to the cavity of the micelle, but it should be ascribed to the 3,4,5-TMEEE benzamide substituents of **1a**–**c**. It could be inferred that there are two main driving forces: one the formation of the hydrogen bond between the carboxylic acid group of NAP and the oxygen atoms of the ethoxy groups; secondly, π–π stacking between NAP and the substituents of **1a**–**c** could be attributed to inclusion complexation. Due to the strong electron-donating alkoxy groups, the benzene ring of 3,4,5-TMEEE benzamide substituent is a strong electron-donor. Therefore, there is an electronic effect between the benzene ring of 3,4,5-TMEEE benzamide substituents and the naphthalene ring of NAP when π–π stacking occurs. Mostly, π–π stacking results in fluorescence quenching [26,27,28,29,30]. Polyphenolic acids such as tannic acids and gallic acid are used as a protein fluorescence quencher because their aromatic rings induces a π–π interaction with the tryptophan residues. These results suggest that the drug molecule is included in the 3,4,5-TMEEE benzamide substituents at the upper rim of the functionalized calix[4]arene.

### 2.3. The Effect of Drug on Micelle Morphology

**Figure 6:**
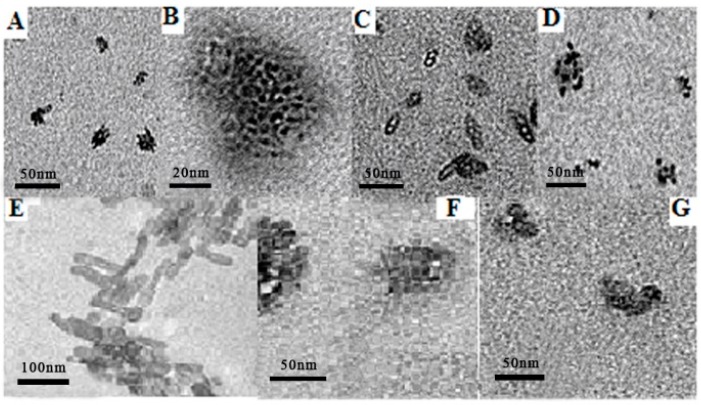
TEM images of (**A**) **1a**; (**B**) HR TEM image of **1a**; (**C**) **1a** + NAP; (**D**) **1a** + IBP; (**E**) **1b**; (**F**) **1b** + NAP; (**G**) **1b** + IBP.

TEM images confirmed that **1a**,**b** form solid micelles in water. When NAP was added into solutions of **1a**,**b** in water, the size of micelles increased considerably, but interestingly, hollow micelles were obtained instead of the anticipated solid micelles (Table 1) (Figure 6). This indicates that NAP was not present in the interior of the micelle, but might have been included in the upper 3,4,5-TMEEE benzamide substituents of **1a**,**b**. The observed shell width of an empty micelle was approximately 4.0 nm, larger than the micelle made of pure **1a**, which has a radius of 2.5 nm. These round blank micelles also have a higher tendency of aggregation and sometimes aggregated into hollow rod-shaped micelles.

Similarly, in the case of **1b**, hollow micelles with a shell width of approximately 5.0 nm were formed, which was much larger than the micelle formed by pure **1b**. These hollow micelles further aggregated into larger nanoparticles. Similarly, when IBP was dissolved into the solution of **1a**,**b** in water, the size of the micelles increased considerably, but were solid, just like those formed by pure **1a**,**b** in water (Table 1) (Figure 6). The resultant micelles were also further aggregated into larger nanoparticles.

**Figure 7:**
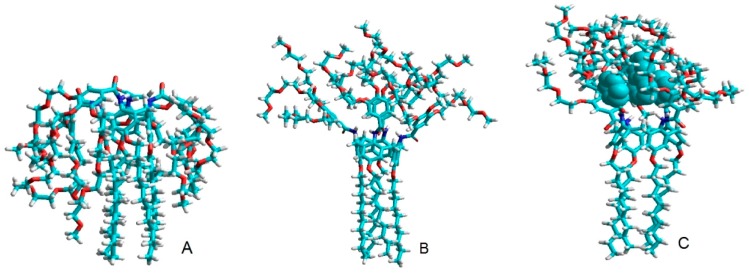
Possible conformers of calixarene **1a** (**A**,**B**) and **1a** + NAP complex (**C**) in water.

In order to understand further the inclusion phenomenon, molecular mechanics calculations were applied for the different conformers of **1a** with and without drug treatment. These calculations showed that, due to their flexibility, the branched 3,4,5-TMEEE benzamide substituents of the calixarene can fold inwards towards the lower rim (Conformer A) (Figure 7) or is stretched over the upper rim of calixarene framework (Conformer B) (Figure 7). Approximating the shapes as rough cuboids, the calculated length/width/height are 2.1 × 1.7 × 2.6 nm for Conformer A and 3.6 × 2.8 × 2.6 nm for Conformer B. Conformer A formed micelles that had a diameter of around 5 nm, so it can be proposed that **1a** exists in a folded conformation as does Conformer A. Conformer A, which was developed as a result of steric repulsive forces and of the hydrophilic nature of the branched substituent groups, has also been reported in previous stuides [23,31,32,33]. After the addition of the drug molecules, the directions of the branched 3,4,5-TMEEE benzamide substituents reversed due to the strong hydrogen bonding between the carboxylic acid groups of the drugs and the oxygen atoms of the 3,4,5-TMEEE benzamide substituents as well as through π–π stacking interactions. The obtained complex of carrier molecules along with the drug as shown in Figure 7 has a dimension of 2.8 × 1.7 × 3.8 nm. This size of complex agrees with the 4 nm shell of the hollow aggregates and the 7.5 nm thickness of the solid micelles (equal to twice the height of Conformer C) when the drug candidates were placed inside them. Conformer C is near-cuboidal in shape and it can form larger blank micelles. These results depict that drug molecule is included in the branched 3,4,5-TMEEE benzamide substituents at the upper rim of the calix[4]arene scaffold instead of the cavity of the micelle.

### 2.4. NMR Investigation

**Figure 8:**
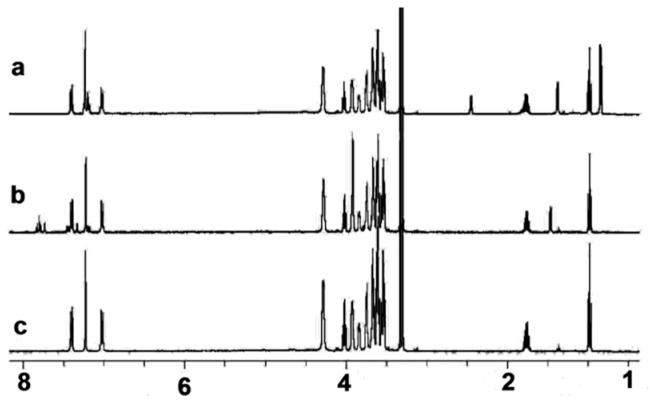
**^1^**H-NMR spectra of (**a**) the **2** + IBP complex, (**b**) the **2** + NAP complex, and (**c**) **2** in D_2_O, [2] = [NAP] = [IBP] = 1.0 × 10^−4^ mol/L in all tests.

NMR investigations were performed in order to further verify the role of the intrinsic cyclic nature of the calix[4]arene in the drug solubilization or inclusion complexation. Firstly, no observable change in chemical shift or broadness in the resonances were observed in the ^1^H-NMR spectra (Figure 8) of the monomeric analogue **2** upon the addition of NAP and/or IBP, thus indicating low or negligible interactions between **2** and NAP and/or IBP.

**Figure 9:**
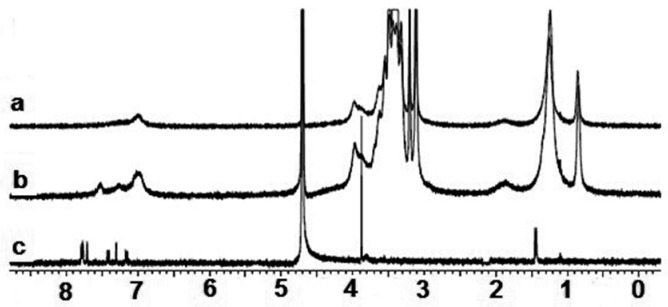
^1^H-NMR spectra of (**a**) **1a**, (**b**) the **1a** + NAP complex, and (**c**) NAP in D_2_O [1a] = [NAP] = 1.0 × 10^−4^ mol/L in all tests.

**Figure 10:**
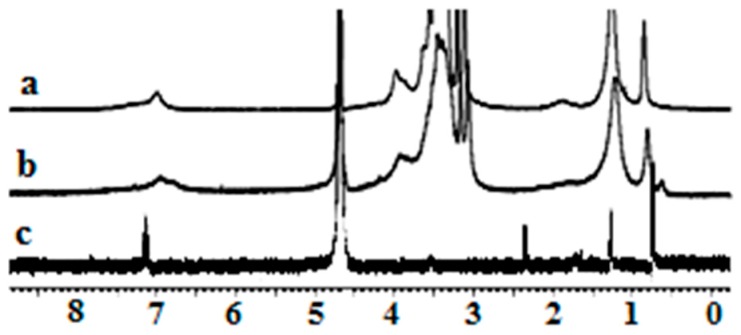
^1^H-NMR spectra of (**a**) **1a**, (**b**) the **1a** + IBP complex, and (**c**) IBP in D_2_O [1a] = [IBP] = 1.0 × 10^−4^ mol/L in all tests.

The ^1^H-NMR analysis of NAP and IBP revealed that all of their NMR peaks became broad as soon as they were mixed with **1a**, indicating ground state non-covalent interactions between **1a** and NAP and/or IBP (Figure 9 and Figure 10). More importantly, the aromatic proton signal at 7.79 ppm of NAP in D_2_O shifted to 7.52 when it was mixed with **1a.** An upfield shift of 0.21 ppm was also observed for the aromatic potons of IBP. NMR titration gave 1:1 inclusion complexes with both NAP and IBP with association constants of 6.3 × 10^4^ M^−1^ and 3.0 × 10^5^ M^−1^, respectively, for the **1a** + NAP and **1a** + IBP complexes. These results support the existance of strong π–π stacking interactions between the aromatic rings of Amphphile **1a** and NAP and/or IBP, and establish the fundamental role of the cyclic nature of the calix[4]arene scaffold in drug solubilization or inclusion complexation.

## 3. Experimental Section

### 3.1. Materials

All reagents and solvents were chemically pure (CP) grade or analytical reagent (AR) grade and were available from Aldrich (Shanghai, China), Acros (Shanghai, China), and Aladdin (Shanghai, China), respectively.

### 3.2. NMR Experiments

^1^H-NMR spectra were measured in CDCl_3_ at 298 K on Bruker 400MHz spectrometer (Bruker, Karlsruhe, Germany) operating at 400.13 MHz. The spectrometer was equipped with a 5 mm Z gradient inversion probe head. The parameter used was 24,038 Hz spectral width for 400 spectrometer. Sixty scans were accumulated using a Bruker sequence. Chemical shifts (CSs) were given using external standard reference (Tetramethylsilane, TMS).

### 3.3. Transmission Electron Microscopy (TEM), HR TEM

Transmission electron micrographs (TEM) were recorded on a G2 20 electron microscope (FEI Co., Eindhoven, North Brabant, The Netherland) at 200 KV. HR TEM was measured with a JEM-2010FEF electron microscope (JOEL Ltd., Tokyo, Japan). The micelle suspension was dropped onto a copper grid covered with a thin carbon film on filter paper and air-dried.

### 3.4. Fluorescence Titration Assay

Fluorescent titration was carried out by the addition of concentrated solution of **1a**–**c** ([1a–c] = 2.0 × 10^−7^–1.0 × 10^−4^ mol/L) and **2** ([2] = 4.0 × 10^−6^–8.0 × 10^−6^ mol/L) into the solution of NAP in H_2_O ([NAP] = 1.0 × 10^−6^ mol/L = constant) by employing λex = 284 nm, em/ex slits = 10/5 nm. To keep a constant concentration of NAP and to account for dilution effects during titration, the solutions of **1a**–**c** and **2** were prepared with a solution of NAP at its initial concentration as a solvent. The quenching constant was calculated using the following Stern–Volmer equation:

F_0_/F = 1 + K_SV_ [Q] (1)

F_0_: fluorescence intensity of NAP; F: fluorescence intensity of NAP upon addition of **1a**–**c**; [Q]: molar concentration (mol/L) of **1a**–**c**; K_SV_: quenching constant (M^−1^).

Steady-State fluorescence spectra were recorded on a Varian Cary Eclipse (Varian Inc., Palo Alto, CA, USA) equipped with a Varian Cary single-cell peltier accessory to control temperature.

### 3.5. Molecular Modeling

All low-energy conformers in aqueous solution were obtained from molecular mechanics calculations by using the MM+ force field implemented in the HyperChem 7.5 program (Hypercube, Inc., Gainesville, FL, USA).

This program was used for the calculations of the calixarene molecules. Different conformers of **1a** with and without drug molecules surrounded by 5000 water molecules were optimized with the molecular mechanics method, respectively.

## 4. Conclusions

We described the synthesis and characterization of a new family of nonionic amphiphilic dendro-calix[4]arene. These compounds increased significantly the solubility of the hydrophobic drugs NAP and IBP in pure water. It was also found that the alkyl chain length and the intrinsic cyclic nature of the calixarene framework are vital for the solubility of the tested drugs. Furthermore, the drug molecules are shown to be included in the branched 3,4,5-TMEEE benzamide substituents of **1a**–**c** at the upper rim of the calixarene rather than in the cavity of the micelle. The drug interacted with **1a**–**c** through hydrogen bonding and π–π stacking. These interactions changed solid micelles of **1a**–**c** into hollow micelles by reversing the direction of the tribranched 3,4,5-TMEEE benzamide substituent of **1a**–**c** from a folded to a stretched state. This finding spurs on new strategies for the design of calixarene-based amphiphilic architectures that can be further used as a nanocarrier for biomedical and pharmaceutical applications.

## Figures and Tables

**Table 1:** Transmission electron microscopy (TEM) of **1a**,**b** with and without NAP and IBP; solubility of NAP and IBP in aqueous solution of **1a**–**c**; quenching constant (K_SV_).

Comp.	TEM Diameter (nm)	TEM Diameter (nm) with NAP	TEM Diameter (nm) with IBP	NAP Solubilty (mg/mL) *	IBP Solubilty (mg/mL) *	Quenching Constant (K_SV_) M^−1^
**1a**	5.0	12.2	10.0	2.10	3.00	5.5 × 10^5^
**1b**	7.1	11.4	08.5	2.50	3.50	5.8 × 10^5^
**1c**				2.90	3.80	6.1 × 10^5^
**2**				0.30	0.60	

* 1.00 mL solution of 10.00 mM **1a**–**c** and **2** could dissolve the corresponding quantities of NAP and IBP, respectively to give a clear solution.

## Supplementary Materials

The following are available online, Scheme S1: Synthesis of architecture **6** (3,4,5-TMEE benzoyl chloride). (a) (CH_3_)_2_SO_4_, NaOH, 120 °C; (b) NaOH aq, TsCl, THF, 0 °C; (c) C_7_H_8_O_5_, KI, K_2_CO_3_, Acetone, reflux; (d) NaOH, H_2_O, reflux; (e) SOCl_2_, reflux, Scheme S2: Synthesis of **10a**–**c**. (a) RBr, NaH, DMF, 85 °C; (b) HNO_3_, CHCOOH, CH_2_Cl_2_, 0 °C; (c) 10%Pd/C, CH_2_Cl_2_, Ethanol, reflux, Scheme S3: Synthesis of compounds **11a**–**c** (Compounds **1a**–**c** in original article), Scheme S4: Synthesis of compound **15** (Compound **2** in original article). Synthesis of surfactants is presented in Supplementary Information.
