# Supplementary material for: Inclusion Complexes of a New Family of Non-Ionic Amphiphilic Dendrocalix[4]arene and Poorly Water-Soluble Drugs Naproxen and Ibuprofen
Source: Molecules. 2017 May 11;22(5):783. doi: 10.3390/molecules22050783 (PMC6154630; doi:10.3390/molecules22050783)
Supplement: Supplementary file 1 [file molecules-22-00783-s001.pdf]

Supporting information

Inclusion Complexes of a New Family of Non-Ionic Amphiphilic Dendrocalix[4]arene and Poorly Water-Soluble Drugs Naproxen and Ibuprofen

Khalid Khan [1,*], Syed Lal Badshah [1], Nasir Ahmad [1], Haroon Ur Rashid [2] and Yahia Mabkhot [3,*]

[1] Department of Chemistry, Islamia College University, Peshawar 25120, Khyber Pukhtunkhwa, Pakistan; shahbiochemist@gmail.com (S.L.B.); nasirshah121@yahoo.com (N.A.)

[2] Department of Chemistry, Sarhad University of Science and Technology, Peshawar 25120, Khyber Pukhtunkhwa, Pakistan; haroongold@gmail.com

[3] Department of Chemistry, College of Science, King Saud University, Riyadh 11495, Saudi Arabia

* Correspondence: drkhalidchem@yahoo.com (K.K.); yahia@ksu.edu.sa (Y.M.); Tel.: +92-312-772-2787 (K.K.); +966-11-467-5898 (Y.M.)

Note: The below synthesis part was published in the article "Khan, K.; Huang, H.; Zheng, Y.-S. Design, Synthesis, and Transport Potential of a New Family of Nonionic Amphiphilic Dendro-calix[4]arene. *Curr. Org. Chem.* **2012**, *16*, 2745–2751." But here we add it again so that it is available to the general public as "Molecule" is an open source journal on the request of reviewers and editor.

Scheme S1

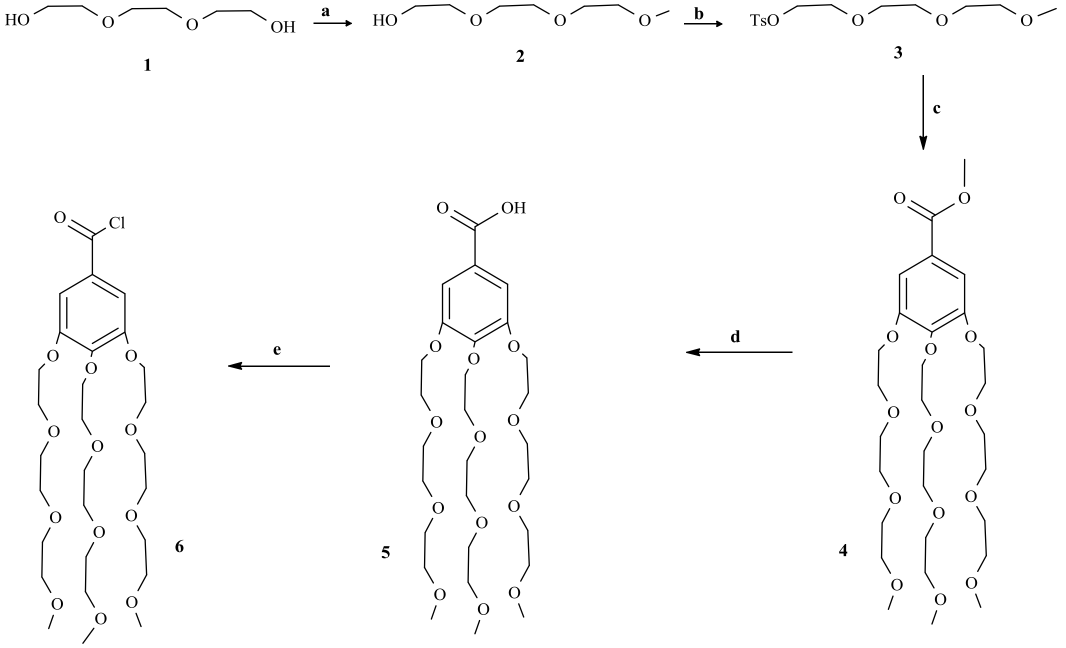

Scheme S1. Synthesis of architecture 6 (3,4,5-TMEE benzoyl chloride). (a) $(CH_3)_2SO_4$, NaOH, 120 °C; (b) NaOH aq, TsCl, THF, 0 °C; (c) $C_7H_8O_5$, KI, K_2CO_3, Acetone, reflux; (d) NaOH, H_2O, reflux; (e) $SOCl_2$, reflux.

Scheme S2

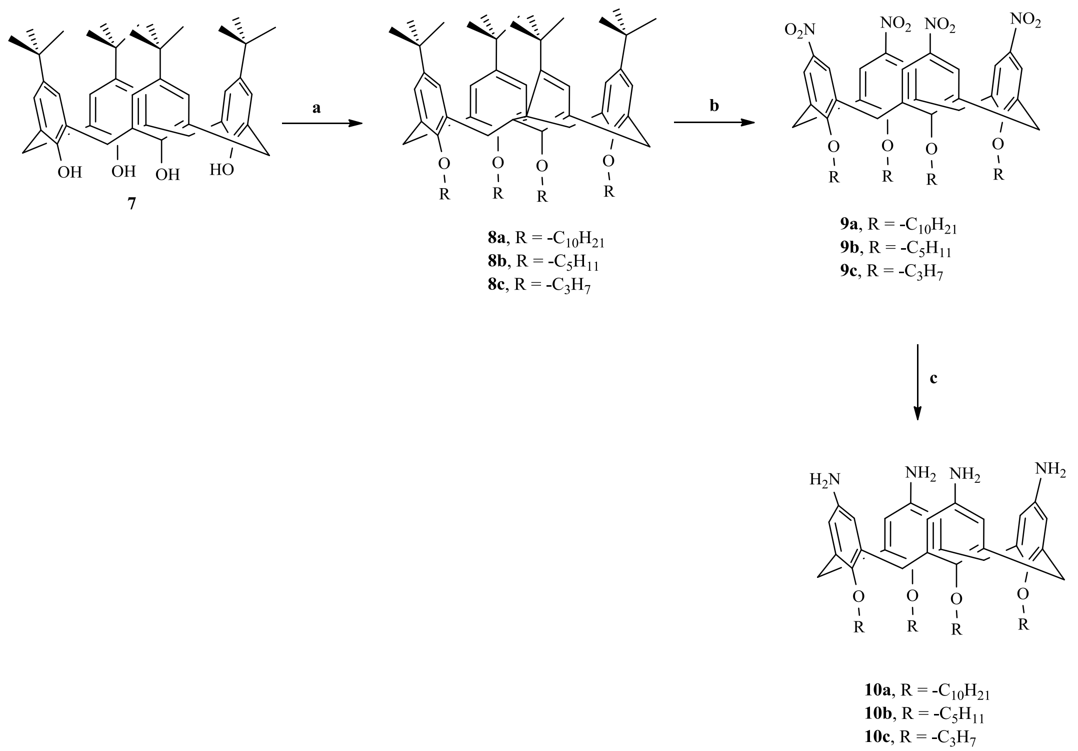

Scheme S2. Synthesis of 10a–c. (a) RBr, NaH, DMF, 85 °C; (b) HNO3, CHCOOH, CH_2Cl_2, 0 °C; (c) 10%Pd/C, CH_2Cl_2, Ethanol, reflux.

Scheme S3

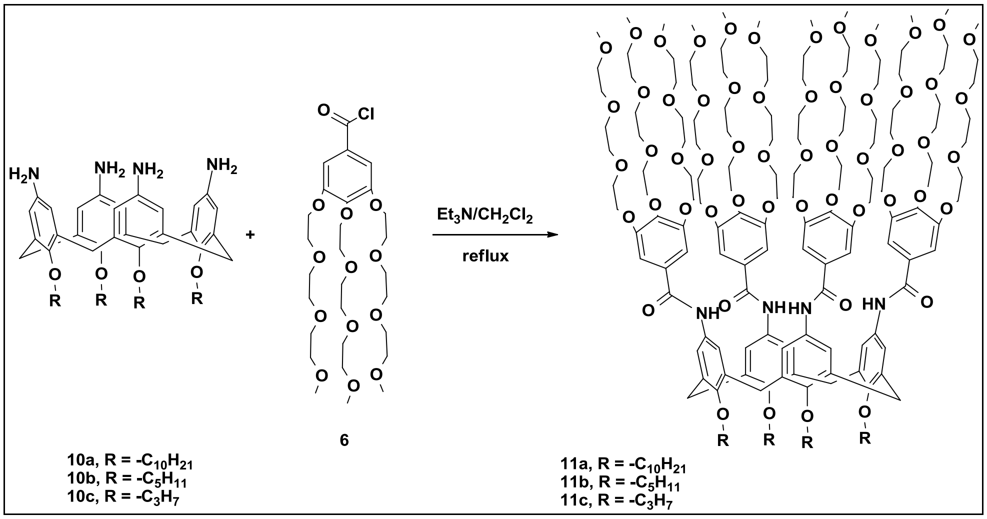

Scheme S3. Synthesis of compounds 11a–c (Compounds 1a–c in original article).

Scheme S4

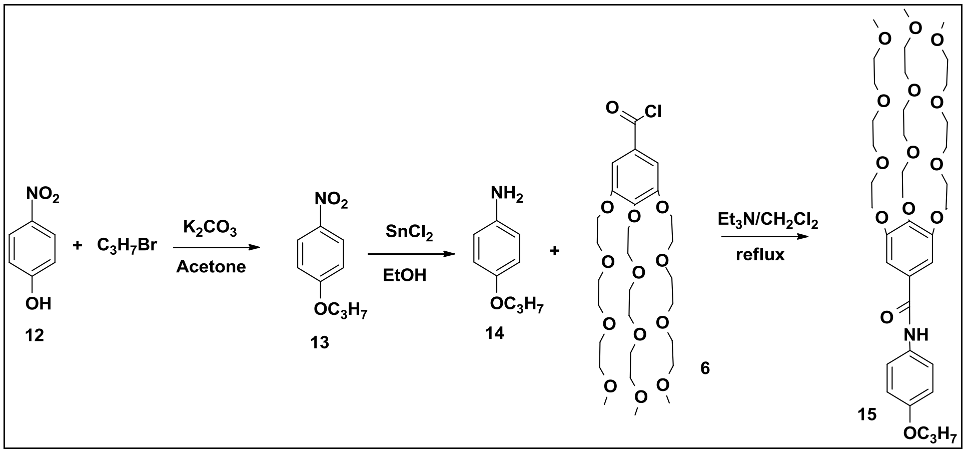

Scheme S4. Synthesis of compound 15 (Compound 2 in original article).

Synthesis of Surfactants

General procedure of synthesis of compounds 11a–c (Compounds 1a–c in Original article): To a solution of **6** (1.0 g, 1.6 mmol) in CH_2Cl_2 (10 mL) cooled with an ice bath was dropped the solution of **10a–c** (0.3 g, 0.29 mmol) and redistilled triethylene amine (Et3N) (0.3 mL, 2.2 mmol) in CH_2Cl_2 (15 mL). After finished addition, the ice bath was removed and the mixture was refluxed for about 3.0 h. The mixture was evaporated to dryness using a rotary evaporator under reduced pressure. The residue was purified by flash column chromatography (silica gel, $CHCl_3/CH_3OH$ 80:1).

[11a=1a]: Colorless or light yellow, yield: (0.45 g, 46%) ^{1}H-NMR (400 MHz, $CDCl_3$) δ 8.26 (s, 4H. CONH), 7.20=7.0 (br s, 16H, ArH), 4.49 (d, *J* = 13.2 Hz, 4H, $ArCH_2Ar$), 4.20–3.40 (m, 152H,CH_2O), 3.36, 3.31 (2s, 36H, OCH_3), 3.21 (D, *J* = 13.2 Hz, 4H, $ArCH_2Ar$), 1.96 (s, 8H, $ArOCH_2CH_2CH_2$), 1.45–1.20 (m, 56H, $OCH_2CH_2(CH_2)_7CH_3$), 0.89 (t, *J* = 6.4 Hz, 12H, CH_2CH_3); ^{13}C-NMR (100 MHz, $CDCl_3$) δ 165.1, 153.5, 152.4, 141.7, 135.2, 132.4, 129.8, 121.3, 107.6, 75.5, 72.4, 72.0, 71.9, 70.68, 70.66, 70.60, 70.56, 70.4, 69.8, 69.2, 59.0, 58.9, 32.0, 31.4, 30.3, 30.0, 29.8, 29.5, 26.4, 22.7, 14.1); IR (KBr) *v* 3308, 2925, 1663, 1584, 1536, 1495, 1468, 1426 cm^{-1}; +TOF HRMS *m*/*z* calcd for $(C_{180}H_{294}N_4O_{56})/2$ 1704.0140 $[M + 2H^+]^{2+}/2$, found $[M + 2H^+]^{2+}/2$ 1704.0179.

[11b=1b]: Colorless or light yellow, yield: (0.65 g, 53%) ^{1}H-NMR (400 MHz, $CDCl_3$) δ 8.41(s, 4H. CONH), 7.20=7.0 (br s, 16H, ArH), 4.50 (d, *J* = 13.2 Hz, 4H, $ArCH_2Ar$), 4.20–3.40 (m, 152H, CH_2O), 3.36, 3.30 (2s, 36H, OCH_3), 3.22 (D, *J* = 13.2 Hz, 4H, $ArCH_2Ar$), 1.97 (s, 8H, $ArOCH_2CH_2CH_2$), 1.41–1.20 (m, 16H, $OCH_2CH_2(CH_2)_2CH_3$), 0.96 (t, *J* = 6.4 Hz, 12H, CH_2CH_3); ^{13}C-NMR (100 MHz, $CDCl_3$) δ 165.0, 153.4, 152.2, 141.4, 135.1, 132.4, 129.8, 121.2, 107.4, 75.4, 72.3, 71.9, 71.8, 70.61, 70.59, 70.54, 70.46, 70.31, 69.69, 69.03, 59.0, 58.84, 31.34, 30.91, 22.81, 14.18; IR (KBr) v 3488, 2927, 1650, 1588, 1543, 1472, 1468, 1427cm^{-1}; +TOF HRMS *m*/*z* calcd for $(C_{160}H_{252}N_4O_{56})/2$ 1562.8497 $[M + 2H^+]^{2+}/2$, found $[M + 2H^+]^{2+}/2$ 1562.1490.

[11c=1c]: Colorless or light yellow, yield: (0.60 g, 42%) ^{1}H-NMR (400 MHz, $CDCl_3$) δ 8.31 (s, 4H. CONH), 7.3=7.1 (br s, 16H, ArH), 4.51 (d, *J* = 13.2 Hz, 4H, $ArCH_2Ar$), 4.22–3.44 (m, 152H, CH_2O), 3.37–3.35 (2s, 36H, OCH_3), 3.29 (D, *J* = 13.2 Hz, 4H, $ArCH_2Ar$), 1.98 (s, 8H, $ArOCH_2CH_2CH_3$), 0.98 (t, *J* = 6.4 Hz, 12H, CH_2CH_3); ^{13}C-NMR (100 MHz, $CDCl_3$) δ 164.7, 153.0, 152.0, 140.35, 135.0, 133.0, 130.0, 121.26, 107.0, 77.83, 77.2, 76.8, 72.05, 71.63, 70.28, 70.18, 70.09, 70.0, 68.5, 60.10, 58.71, 58.63, 31.11, 14.03 ; IR (KBr) v 3443, 2925, 1648, 1588, 1542, 1468, 1427 cm^{-1}; +TOF HRMS *m*/*z* calcd for $(C_{152}H_{236}N_4O_{56})/2$ 1506.7870 $[M + 2H^+]^{2+}/2$, found $[M + 2H^+]^{2+}/2$ 1506.0871.

3.2. Synthesis of Compound 13 (Compound 15=2 in Original Aricle)

Reaction conditions and workup were the same as described above for the **11a–c**, while had different molar ratio with **6** (2.5 g, 4 mmol) and **12** (0.50 g, 3.3 mmol). Light yellow, yield: (2 g, 80%) ^{1}H-NMR (400 MHz, $CDCl_3$) δ 8.22 (s, 1H. CONH), 7.57 (d, *J* = 8.8 Hz, 2H, ArH), 7.28=7.20 (br s, 2H, Ar), 6.8 (d, *J* = 8.8 Hz, 2H, ArH), 4.19–3.90 (m, 38H, CH_2O), 3.37–3.32 (2s, 9H, OCH_3), 1.8 (s, 2H, $ArOCH_2CH_2CH_3$), 1.03 (t, *J* = 7.2 Hz, 3H, CH_2CH_3); ^{13}C-NMR (100 MHz, $CDCl_3$) δ

165.29, 155.96, 152.47, 141.65, 131.33, 130.23, 122.22, 114.71, 107.67, 77.07, 76.75, 72.36, 71.93, 71.88, 70.61, 70.55, 70.50, 70.43, 69.78, 69.13, 58.98, 58.91, 22.60, 10.50; IR (KBr) ν 3478, 2928, 1650, 1587, 1508, 1460, 1427 ; +TOF HRMS *m*/*z* calcd for ($C_{37}H_{59}NO_{14}$)/2 370.93133 [M + 2H$^+$]$^{2+}$/2, found [M + 2H$^+$]$^{2+}$/2 370.69678.
